# Diving accidents: a cohort study from the Netherlands

**DOI:** 10.1186/s12245-016-0109-4

**Published:** 2016-03-12

**Authors:** J. W. Smithuis, E. Gips, T. P. van Rees Vellinga, M. I. Gaakeer

**Affiliations:** Department of Emergency Medicine, VU University Medical Center, Amsterdam, Netherlands; Department of Emergency Medicine, Fiona Stanley Hospital, Murdoch, Perth, Western Australia; Medical Centre Hyperbaric Oxygen Therapy (MCHZ), Goes, Netherlands; Department of Emergency Medicine, Admiraal De Ruyter Hospital, Goes, Netherlands

## Abstract

**Background:**

Diving is, besides professional reasons, an increasingly popular leisure activity. Whilst statistically compared to other sports safe, diving accidents can result in serious complications. In order to treat this specific patient category adequately, early diagnosis is important. In this study, we explore various medical aspects of diving accidents. By sharing our experiences, we intend to create awareness and enhance urgent medical care for this specific category of patients.

**Methods:**

We conducted a retrospective cohort study using anonymized patient records from the emergency department (ED) of the Admiraal De Ruyter Hospital (ADRZ) and affiliated Medical Centre Hyperbaric Oxygen Therapy (MCHZ1) both in Goes, Netherlands. We evaluated all patients that presented to our ED as a diving accident from 1 November 2011 to 30 August 2015.

**Results:**

In the selected period, 43 patients presented to our ED with complaints after diving; 84 % were male and 49 % older than 40 years, and they came by ambulance or referred by a general practitioner or other medical centres in the area; 70 % presented the same date as their dive, 21 % 1 to 3 days and 9 % later than 3 days after having dived. Pain was the most frequently reported symptom (44 %), followed by constitutional symptoms (42 %). Numbness or paraesthesia was reported in 33 %. Respiratory symptoms, dizziness, a change in mental status (e.g. apathy, confused or restlessness) and problems with coordination were present in 10–21 % of the cases. Symptoms that were apparent in less than 10 % of the cases were cutis marmorata, visual or auditory complaints, muscle weakness, cardiovascular symptoms or a malfunction of the anal sphincter or urinary bladder. Most of our patients exhibited more than one symptom; 70 % of all patients received hyperbaric oxygen recompression therapy.

**Conclusions:**

The limited number of patients presenting with complaints after a diving incident, the difficulty of recognition and the (potential) huge impact if not recognized and treated adequately make us believe that every diving accident should be discussed with a centre of expertise.

## Background

### Introduction

Diving is, besides for professional reasons, a popular leisure activity showing a steady growth of certified divers for the last 10 years [1]. Whilst statistically compared to other sports safe, in general, diving is considered an adventurous and risky sport. Every dive entails potential risks ranging from minor injuries to life-threatening situations. The death rate among US recreational divers is estimated at 3–6 per 100,000 [2]. Diving accidents can result from a variety of causes, including physical exhaustion, hypothermia, pre-existing disease, poor buoyancy control with rapid ascent, risk behaviour, (miscalculation of) hazardous conditions like violent water movement or poor visibility and failure of technique.

### Importance

Diving accidents can result in serious complications, such as decompression sickness (DCS), barotrauma and arterial gas embolism. Because of the potential seriousness of these disorders, adequate treatment is important. Hyperbaric oxygen is the main therapy for DCS by decreasing bubble size and counteracting anti-inflammatory responses [3–5]. Urgent decompression is advised, although even in substantially delayed presentation treatment is effective [6–8].

### Goals of this investigation

The main objective of the present study is to provide a detailed description of the types, symptoms and outcomes of diving accidents presented to our emergency department (ED) in the Zeeland Delta, Netherlands. By sharing our experiences, we intend to create awareness and enhance medical care for this group of patients.

## Methods

### Study design

We conducted a retrospective cohort study using anonymized patient records from the ED and affiliated Medical Centre Hyperbaric Oxygen Therapy (MCHZ).

### Setting

In 1953, a terrible flood devastated the province of Zeeland. A coastal defence in the form of the Delta Works was built for protection against future flooding. This huge artificial reef supports unique marine life, making it a very popular dive destination.

This study is performed at the ED of the Admiraal De Ruyter Hospital (ADRZ), which also houses the MCHZ. The ADRZ is a community hospital and houses the only 24/7 available Level II Trauma Centre ED in the Zeeland Delta. Since 1 November 2011, treatment of diving accidents using hyperbaric oxygen therapy (HBOT) is possible. A multidisciplinary team, including an emergency physician (EP) and a doctor of hyperbaric medicine, is always available.

### Participants and data sources

We included all diving accidents presenting to our ED and MCHZ from November 2011 to September 2015. Data collection was performed from March 2015 to November 2015 by JS. This involved looking up hand-written records from the MCHZ and digitalized reports from the ED. Furthermore, patient records were requested from affiliated medical centres if included patients were transported for treatment elsewhere. Information from included patient records was organized in Excel and analysed by two authors (JS and MIG). Inter-observer agreement was calculated by the Cohen *κ* statistic using the variable ‘constitutional symptoms’. The GraphPad Prism software has been used for statistical computing.

### Variables

The data from medical records included age, sex, maximal depth, dive duration, reported and presenting symptoms, given therapy, decompression stops during ascend, time from surfacing to recompression and treatment outcome.

We classified symptoms using organ systems (e.g., auditory, pulmonary) if possible. For less well-defined complaints, we sorted symptoms as reported (e.g., dizziness, pain). This classification is based on the system used in a previous publication [9]. If any patient record was incomplete for some of the data, this is clearly stated in this paper.

## Results

### General results

**Fig. 1:**
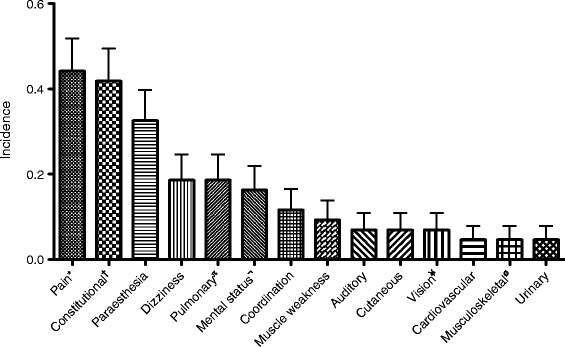
Distribution of presenting symptoms. Pain consisted of joint pain (56 %), abdominal pain (22 %), muscle pain (11 %) or pain somewhere else (17 %) (*asterisk*). Constitutional symptoms included tiredness, light-headedness, fatigue, nausea and vomiting (*dagger*). Pulmonary symptoms included dyspnoea, coughing and pain during respiration (*Pi*). Dullness, mental confusion and a loss of concentration were signs of mental symptoms (not sign). Symptoms of vision involved blurry vision and diplopia (*Yen*). Musculoskeletal symptoms included muscle cramps and stiffness (l*etter O with stroke*)

**Fig. 2:**
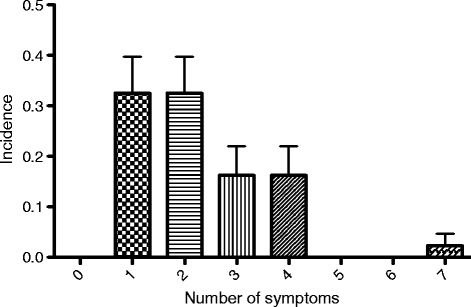
Incidence of amount of symptoms

In the study period, 43 patients presented with complaints after diving. Patients came via ambulance or were referred by a general practitioner or other medical centres in the area. Demographic data of our study population is grouped in Table 1. A vast majority of the divers were men (84 %). A relatively large number were more than 40 years of age (49 %). Interestingly, many patients (that we know of) did make a decompression stop during their ascend (42 %). Most patients presented to our hospital on the same day as their dive. However, three patients (5 %) presented later than 3 days after having dived. Of these patients, only one had severe complaints (diplopia), whereas the other two patients reported mild paraesthesia or musculoskeletal pain. He was treated with hyperbaric oxygen, despite the late presentation, following a complete relief of his visual symptoms. Figure 1 gives an overview of the symptoms our patients presented with. Pain was the most frequently reported symptom (44 %), followed by constitutional symptoms (42 %). Pain was mostly located in the joints (56 %). Other reported locations of pain included the muscles, face, and abdomen. Another frequently reported complaint was numbness or paraesthesia (33 %). In most patients, this was located in the extremities. Respiratory symptoms, dizziness, a change in mental status (e.g. apathy, confusion or restlessness) and problems with coordination were symptoms present in 10–21 % of the cases. Symptoms that were apparent in less than 10 % of the cases were cutis marmorata, visual or auditory complaints, muscle weakness, cardiovascular symptoms or a malfunction of the anal sphincter or urinary bladder. Inter-observer agreement for assigning constitutional symptoms was substantial *κ* = 0.76 (95 % CI = 0.57–0.96). Most of our patients exhibited more than one symptom. Using the same symptom classification as the previous figure, Fig. 2 shows the incidence of patients presenting with a various amount of symptoms. Of our patients, 33 % showed only one symptom. The same number of patients had two symptoms; 16 % had three symptoms, the same number of patients had four symptoms. One of our patients (*case D*) had seven symptoms. Not all diving accidents received HBOT, but 70 % of the patients did. Of these patients treated, 76 % were completely symptom free afterwards, and 14 % reported persistent complaints after therapy; in 10 %, these results could not be determined from the patient records.

**Table 1:** Demographic data of our study population

Category	Variable	Patients, *N* (%) (*n* = 43)
Gender	Male	36 (84)
	Female	7 (16)
Age groups (years)	0–20	2 (5)
	20–40	20 (47)
	40–60	16 (37)
	60–80	5 (12)
Decompression stop during ascent	Yes	18 (42)
	No	14 (33)
	Unknown	11 (26)
Time between dive and hospital visit (days)	0	30 (70)
	1–3	10 (23)
	>3	3 (5)

### Illustrative cases

*Patient A*, a 29-year-old female, had dived for 28 min at a maximum depth of 21 m and had surfaced without a decompression safety stop. She felt a ‘snap’ and a sudden pain in the left thorax during ascent, after which she experienced dyspnoea. After reaching the surface, she also suffered paralysis of the right leg and paraesthesia in both legs. Physical examination in the ED revealed tachypnoea (25/min), an oxygen saturation of 100 % receiving 15 L O_2_ with a non-rebreather mask (NRM) and decreased breath sounds over the left thorax. The main neurologic finding was ataxia of the right leg. Chest X-ray demonstrated a left-sided pneumothorax. Additional imaging with computed tomography (CT) revealed pre-existing bullae of the left lung (a risk factor for barotrauma during diving). Patient A was diagnosed with a spinal artery gas embolism caused by a barotrauma with a pneumothorax. She was treated with hyperbaric oxygen after insertion of a chest drain. She recovered fully following these treatments.

**Fig. 3:**
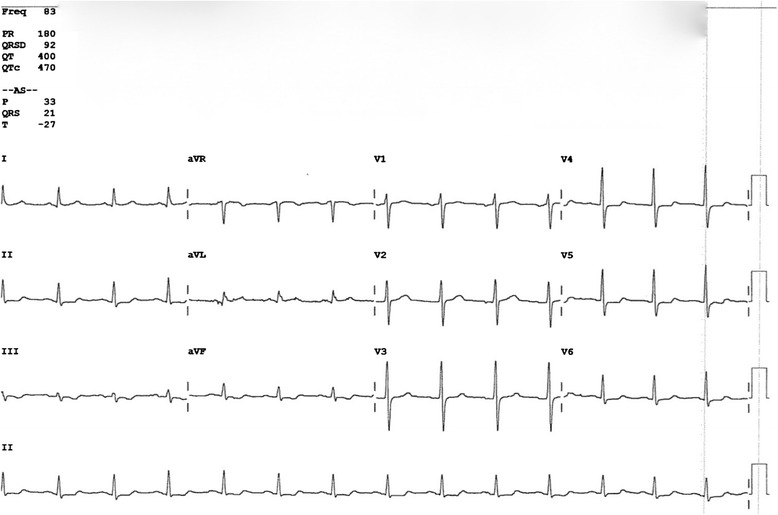
EKG of patient B

*Patient B*, a 51-year-old male, had dived for 20 min at a maximum depth of 40 m and made a rapid ascent from 30 m depth without a decompression stop after experiencing a pressing pain on his chest. At the surface, he experienced dyspnoea, persisting thoracic pain and paraesthesia in his right arm and leg. These symptoms were still present upon arrival in our ED. The EKG (Fig. 3) revealed signs of an inferior-lateral myocardial infarct showing ST depression in leads aVF, V4 and V5. His highly sensitive troponin level was elevated (1.3 μg/L). Patient B was treated with recompression therapy under suspicion of a coronary arterial gas embolus. Following HBOT, a coronary angiography was performed, showing normal coronary arteries. His chest symptoms resolved and troponin levels and EKG findings returned to normal after several days.

*Patient C*, a 54-year-old male, had dived for 44 min at a maximum depth of 44 m and ascended performing the determined decompression stops. The dive went according to plan. Approximately 14 h after diving, he developed the inability to urinate and difficulty walking. In the ED, physical examination showed a paralysis of the right leg, Babinski sign was positive bilaterally and there was a loss of the anal sphincter function. Bladder scanning showed >1000 ml volume, consistent with urinary retention. MRI of the lumbar spine did not show a clear cause for the symptoms. He was diagnosed with transient paraplegia caused by a spinal artery gas embolism. Following 3 days of HBOT, motor function of the legs, urinary control and reflexes of the feet and anal sphincter returned to normal.

**Fig. 4:**
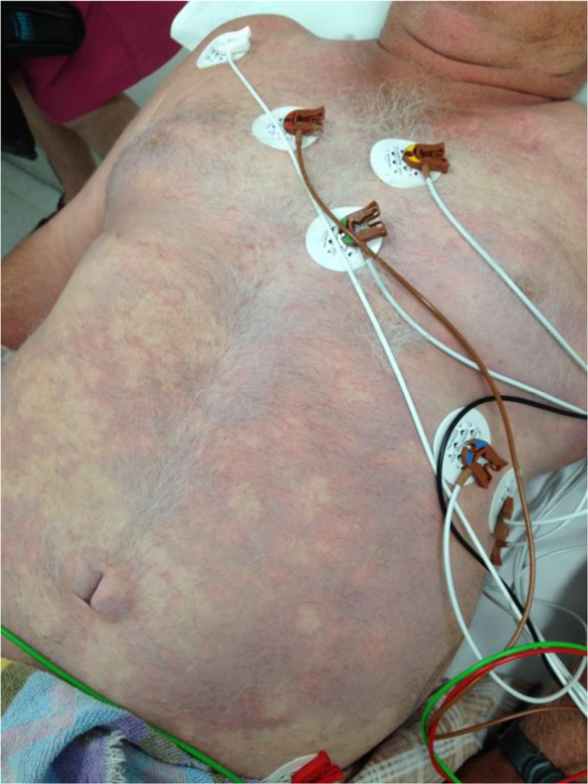
Cutis marmorata. This picture (not belonging to patient D) shows extensive cutis marmorata, a symptom of decompression illness. Source: Dr. Neil Banham MBBS, FACEM, Dip DHM, Head of Department Hyperbaric Medicine Unit, Fiona Stanley Hospital, Australia

*Patient D*, a 54-year-old male, had dived for 40 min at a maximum depth of 46 m. Because he ran out of air, he made an emergency ascent from 46 m without using decompression stops. In our ED, he was confused and restless. He was tachypnoeic, pulse oximetry showed an oxygen saturation of 90 %, whilst receiving supplemental oxygen with a NRM. He had a contracture of his neck towards the left side as well as a dilated left pupil and a loss of motor function of his left leg. Furthermore, his abdomen showed a rash consistent with cutis marmorata (Fig. 4). After decompression, the restlessness and disorientation worsened after which the patient was sedated with propofol. After decompression, the patient remained disoriented, but was not dyspnoeic anymore and showed normal pupillary reactions. However, he developed a triparesis (both legs and left arm), his left lower leg showed signs of ischemia and he established acute renal failure. For further treatment, this patient was transferred to the nearest academic centre, where the patient was intubated and treated with serial hyperbaric oxygen therapy. Imaging showed a diffuse oedema of the cervical and thoracic cord. Trans-thoracic echocardiogram (TTE) revealed a patent foramen ovale (PFO), which is a recognized risk factor for arterial gas embolism during diving [10]. At the time of writing, the patient still resided in a rehabilitation centre. His cognition was fully recovered and he was able to walk very short distances using a walker.

*Patient E*, a 48-year-old male, had dived for 30 min at a maximum depth of 17 m. At 17 m, he felt lightheaded, confused and lacked control over his movements. Exhibiting these complaints, he ascended in a controlled way. In our ED, primary survey did not show any abnormalities. However, laboratory testing showed a carbon monoxide (CO) level of 13.6 %. After decompression therapy, the patient was asymptomatic. Further investigation as to the cause of the CO intoxication in cooperation with the police department revealed a batch of compression tanks with likely a contaminated fill.

## Discussion

In this publication, we have given an overview of the diving accidents in the Netherlands presented to our ED. Our data show that the number of diving accidents presenting is rather limited, 43 in a period of more than three and a half years, especially when one takes into account the estimated 800,000 recreational dives just in the Zeeland Delta each year [11]. Diving accidents presented with a wide variety of symptoms ranging from a mild musculoskeletal pain to time-critical neurological and cardiac disorders. The incidence of the symptoms in our cohort seems to be similar to a bigger cohort of 2346 divers previously described in the Lancet [9]. The wide spectrum of symptoms can make it challenging for any doctor to attribute the complaints to decompression sickness or arterial gas embolism, especially as symptoms may not start on the day of the dive. Also, the frequent coexistence of two or more complaints can make it hard to diagnose DCI. However, it is essential to establish rapidly whether medical complaints are dive related or not and if recompression therapy is indicated. HBOT is accepted worldwide as the appropriate treatment for DCI with a reduction of recovery time and an improvement of outcome. Of all diving accidents presenting to our ED in the study period, 70 % received hyperbaric oxygen recompression therapy. Reasons not to administer hyperbaric oxygen were mainly because of absence of signs and symptoms at the moment of ED presentation or the presence of mild complaints only (21 %). Other reported reasons not to treat divers with recompression therapy were significant delay between the dive and onset of symptoms or because presenting symptoms were thought not likely to be related to the dive. Assessment and initial management of a patient that has suffered a diving accident should first be to adhere to basic and advanced trauma and life support principles with a thorough primary and secondary survey in order not to miss another possible acute diagnosis or additional relevant traumatic injury. For example, a pneumothorax has to be firmly excluded before hyperbaric treatment is started. In order to make a quick assessment of the seriousness of the dive accident, a good patient history should then be taken concerning dive depth, duration and whether there has been done any safety or decompression stops during ascent. However, as our data suggest, even making decompression stops does not exclude the possibility of DCI.

## Conclusion

Even though we have been able to include a significant amount of patients, our numbers limit us in composing firm conclusions. Nevertheless, the limited number of patients presenting with complaints after a diving incident, the difficulty of symptom recognition and the (potential) huge impact if not recognized and treated adequately make us believe that every diving accident should be discussed with a centre of expertise.

### Limitations

This research has been conducted using the data of our ED and the MCHZ. Our ED is the only facility in the vicinity of the seashore in this region of the Netherlands. Therefore, we were only able to give an image of diving accidents of our region. Our hospital hosts the only local 24/7 hyperbaric treatment centre. Because our community ED is a level 2 trauma centre, we were unable to perform a thorough follow-up of severe dive accident patients that had to be transferred to an academic medical centre, mostly after their first decompression therapy in our hospital. Patients that died at the diving scene and did not present to our ED were not included in this study.
